# Inference of time-delayed gene regulatory networks based on dynamic Bayesian network hybrid learning method

**DOI:** 10.18632/oncotarget.21268

**Published:** 2017-09-23

**Authors:** Bin Yu, Jia-Meng Xu, Shan Li, Cheng Chen, Rui-Xin Chen, Lei Wang, Yan Zhang, Ming-Hui Wang

**Affiliations:** ^1^ College of Mathematics and Physics, Qingdao University of Science and Technology, Qingdao 266061, China; ^2^ CAS Key Laboratory of Geospace Environment, Department of Geophysics and Planetary Science, University of Science and Technology of China, Hefei 230026, China; ^3^ Bioinformatics and Systems Biology Research Center, Qingdao University of Science and Technology, Qingdao 266061, China; ^4^ Key Laboratory of Eco-chemical Engineering, Ministry of Education, Laboratory of Inorganic Synthesis and Applied Chemistry, College of Chemistry and Molecular Engineering, Qingdao University of Science and Technology, Qingdao 266042, China; ^5^ College of Electromechanical Engineering, Qingdao University of Science and Technology, Qingdao 266061, China

**Keywords:** gene regulatory networks, multiple time-delayed, dynamic Bayesian network, comprehensive score model, network structure profiles

## Abstract

Gene regulatory networks (GRNs) research reveals complex life phenomena from the perspective of gene interaction, which is an important research field in systems biology. Traditional Bayesian networks have a high computational complexity, and the network structure scoring model has a single feature. Information-based approaches cannot identify the direction of regulation. In order to make up for the shortcomings of the above methods, this paper presents a novel hybrid learning method (DBNCS) based on dynamic Bayesian network (DBN) to construct the multiple time-delayed GRNs for the first time, combining the comprehensive score (CS) with the DBN model. DBNCS algorithm first uses CMI2NI (conditional mutual inclusive information-based network inference) algorithm for network structure profiles learning, namely the construction of search space. Then the redundant regulations are removed by using the recursive optimization algorithm (RO), thereby reduce the false positive rate. Secondly, the network structure profiles are decomposed into a set of cliques without loss, which can significantly reduce the computational complexity. Finally, DBN model is used to identify the direction of gene regulation within the cliques and search for the optimal network structure. The performance of DBNCS algorithm is evaluated by the benchmark GRN datasets from DREAM challenge as well as the SOS DNA repair network in *Escherichia coli*, and compared with other state-of-the-art methods. The experimental results show the rationality of the algorithm design and the outstanding performance of the GRNs.

## INTRODUCTION

Gene regulatory networks (GRNs) is the mechanism of gene expression *in vivo* of biology, and dominates the various physiological activities of the organism [1–4]. The application of gene chip technology in system biology provides a great deal of basic data for GRNs research and analysis. As a research frontier in systems biology, the GRNs tries to excavate regulatory networks from gene expression data from the system point of view, revealing complex biological phenomena from the perspective of gene interactions [5–8]. The study of GRNs has a very important biological significance. According to the topological structure of regulatory networks, it is possible to know the influence of various regulatory relations on gene expression level and to provide a possibility for more in-depth understanding of gene transcription, process of translation mechanism, disease formation, drug design, and reverse engineering [9].

From the perspective of machine learning, there are two ways to construct GRNs: supervised learning method and unsupervised learning method [10–15]. Supervised learning refers to the need to construct a network with prior knowledge, such as regulatory factor data and regulatory relational data. Unsupervised learning method refers to the network construction process which does not require known regulatory data, only DNA microarray data (gene expression data) on the basis of the regulation of the network derivation or regulatory relationship prediction. From the results of the prediction, the supervised learning method has stronger ability of discovery, and many experimental results also confirm this viewpoint [16]. However, supervised learning requires a large amount of prior knowledge to be added. Especially, knowledge about topological sorting of nodes is very important for network construction. In practice, this knowledge is often difficult to obtain directly. The method is also divided into two categories: one is based on the model construction method, one is the statistical inference method. The model-based approach focuses on the accurate description of the regulatory network, while the statistical inference method focuses on the quantification of causal strength [17]. Although they are divided into two categories, these two methods are not completely overlapping.

As far as unsupervised learning methods are concerned, Boolean networks [18–21] and differential equation [22–24] are considered to be two extremes of the learning method: Boolean networks are rough qualitative methods, and differential equations use refined mathematical equations to describe the regulatory process. The Bayesian approach is seen as a compromise between the two approaches. Bayesian network (BN) is a theory of describing the probability relation through the directed acyclic graph (DAG). The relationship between the variables is described by the joint probability distribution [25–28]. It is composed of the following two elements: the network topology, which is the directed edge between the nodes and nodes in the graph. The network parameters, that is, the conditional probability table (CPT) of each node represents the dependency of variables. The Bayesian network has the advantages of flexibility, natural integration of prior knowledge, learning causal and other types of relationships, and the ability to handle incomplete data.

At present, the BN structure learning algorithm is divided into two categories [29–31]: score-search based method and constraint-based method. The score-search method focuses on discovering the network structure consistent with the training data to facilitate inference. While the constraint method focuses on the network nodes relationship, which is related to the network derivation process of the constraint method. The GRNs deduced by the constraint method has a causal explanation, which is more consistent with the purpose of GRNs construction. Constraint-based method combined with search algorithm to find the optimal network structure is called hybrid learning method, which is the mainstream method of BN structure learning. BNs are classified into static Bayesian networks (BNs) and dynamic Bayesian networks (DBNs), depending on the type of gene expression data being processed. The static BNs is suitable for dealing with the gene expression data without timing information. The DBN method is suitable for processing gene expression data with time-series information [32]. DBNs is an extension of the standard BN, which combines the causal relationship of time and the causal relationship of variables for simulation and dynamic causal analysis of multivariable stochastic processes. DBNs, like the standard BN, is also composed of two parts: structure and parameter. According to the network structure and dataset, DBN can estimate the parameters, so structure learning is the core of DBN learning. Under stationary and Markovian assumptions, DBN structure learning transforms into prior network and transition network learning problems. At present, DBN structure depends mainly on expert knowledge [33, 34] and scoring-search method [35]. The first method is difficult to work for more variables and the DBN structures got by different experts are often different, which is hard to communicate, compare and analyze. The second method has a good theoretical basis, but the computational complexity of the scoring function and the size of the structure search space increase exponentially with the increment of the variable, so it is generally required that the nodes have order, and adopt greedy or stochastic search methods for structural learning.

In 1998, Friedman et al. [36] extended the static Bayesian scoring model to the dynamic state for the first time, and discussed the general methods of DBN structure learning under complete data and incomplete data, and proposed learning DBN structure algorithm DPN-SEM under incomplete data. The basic idea is to search the network structure with the optimal score in the candidate space. In the process of reconstructing GRNs using BNs, the use of mutual information (MI) can reduce the computational complexity. MI has been widely used to reconstruct GRNs because it provides a natural generalization of the correlation owing to its capability of characterizing non-linear dependency. Furthermore, MI is able to deal with thousands of variables (genes) in the presence of a limited number of samples. However, the MI can only predict the relationship between pairs of genes, and cannot be a gene by a number of genes in the case of regulation [37–39]. Conditional mutual information (CMI) is considered to be a clever complement of MI, which can detect multiple genes co-regulating the target gene regulation model by quantifying the correlation under the condition of co-regulators, so that CMI can be better improved in efficiency, and solve the problem of causal intensity calculation under multiple gene regulation [40]. However, CMI also tends to underestimate the intensity of regulation between variables in some cases [41–43]. A method called tuned mutual information (TMI) is used to make up for the deficiency of CMI. TMI is added to the Kullback-Leibler (KL) divergence between variables on the basis of non-linear correlation information of mining gene expression data, which can theoretically further reduce the false positive rate in the network construction and avoid the unreasonable results caused by the special case when MI is used. For example, Zhang et al. [44] successfully overcame the shortcomings of CMI computation based on the topology of GRN constructed by the path consistency algorithm (PCA) combined with TMI. However, with the influence of TMI characteristics, in addition to the method of modifying the threshold parameters, the algorithm derives the true positive rate of the regulatory network which is difficult to improve through other methods.

In order to reduce the computational complexity, the number of regulatory genes is restricted, the parallel computer is used, and the algorithm itself is improved. Grzegorczyk et al. [45] proposed an inter-gene information sharing method to harmonize the complexity of the automatic model, but the method lacks the inference of non-linear dependency between genes. Adabor et al. [46] proposed a hybrid search algorithm combining greedy algorithm with simulated annealing, which can quickly converge to the local optimal network without restricting the number of nodes. It can save the searching process while ensuring the prediction regulatory network quality. Butte et al. [47] proposed a two-dimensional histogram instead of the joint probability of gene pairs, in order to achieve fast algorithm of MI between genes, but ignoring the dynamic characteristics of gene expression data.

In addition to computational complexity and other issues in the network construction process to hinder the GRN inference from gene expression data, and infer GRN on the accuracy of the results, there are still a large number of unresolved problems. In order to reduce false positives and improve the accuracy of GRN construction, Altay et al. [48] proposed the C3NET algorithm to select the most significant connection of each gene as the edge of the GRN, and then use unsupervised learning algorithm ARACNE to construct GRN. When using C3NET algorithm, the threshold of independence can be determined by human. If the threshold is too high, it will eliminate the potential correct regulation relationship. If the threshold is too low, it will make the derived network have higher false positives. Liu et al. [49] proposed LBN (local Bayesian network) method to reconstruct GRNs. By iteratively performing CMI and BN with kNN methods, LBN method can infer the optimal GRN structure. The inferred network is a static network, which lacks the dynamic feature mining in gene expression data, and cannot infer dynamic GRN.

In the comparison of algorithms for GRNs, Frank et al. [50] analyzed and compared gene regulation construction algorithms in terms of statistical inference. According to the statistics, the statistical inference method is divided into two categories based on correlation and mutual information. The basic idea of statistical inference method to construct a GRN is to calculate the correlation or mutual information between each pair of genes, and to set the threshold to screen the significant gene pairs and control the sparsity of the network. The method of statistical inference has been studied by Schaffter et al. [51]. Six different unsupervised methods have been compared in the simulated datasets, and the z-score method has been found to be the best in the knock-out experiment. Faith et al. [52] evaluated the Context Likelihood of Relatedness (CLR) algorithm for the reconstruction of accurate cellular networks (ARACNE), relevance networks (RN) and a Bayesian network (BN) on an *Escherichia coli* benchmark data set and found that the CLR method performence best. It is worth mentioning that the Dialogue for Reverse Engineering Assessments and Methods (DREAM) challenge [53–55] produced a number of GRN inference methods. The DREAM competition also shows that GRN inference is a very challenging problem.

Traditional BNs have high computational complexity, which are slow to construct large and medium-sized GRNs, and usually have unsatisfactory results [56–59]. The network structure scoring model is single. Information theory-based approach can only determine the independence of variables, which cannot identify the direction of regulation. In order to compensate for the shortcomings of the above methods, we propose a novel hybrid learning algorithm (DBNCS) based on DBN to construct the multiple time-delayed GRNs, by combining comprehensive score (CS) model with DBN model. The CMI2NI algorithm for network structure profiles learning, namely the construction of search space. Using the recursive optimization algorithm (RO) to remove redundant regulations, thereby reduce the false positive rate. CMI2 is used to calculate the optimal transcription time-delayed between pairs of genes in the search space. After the network structure profiles are decomposed into multiple cliques without loss, the DBN model is used to identify the direction of gene regulation within cliques, and the optimal network structure search is carried out, which significantly reduces the computational complexity. The proposed network inference method DBNCS can not only excavates the dynamic information in gene expression data, makes the construction of GRNs more in line with the biological basic physiological mechanism, but also through RO algorithm to mine the linear correlation between genes in gene expression data, through CMI2 mining non-linear correlation, to achieve accurate quantify causal intensity between genes, and effectively improve the accuracy of GRN inference from gene expression data. On the benchmark GRNs from DREAM challenge and a real SOS DNA repair network in *Escherichia coli*, the experimental results show that our method achieves the satisfactory performance in comparision with other existing methods in terms of false positives and accuracy.

## RESULTS AND DISCUSSION

### Network reconstruction on DREAM3 challenge simulation data

In this work, we used DREAM3 challenge two simulation datasets with network sizes 10 and 50 to construct the GRNs by DBNCS algorithm.

### DREAM3 challenge 10 nodes GRNs construction

**Figure 1:**
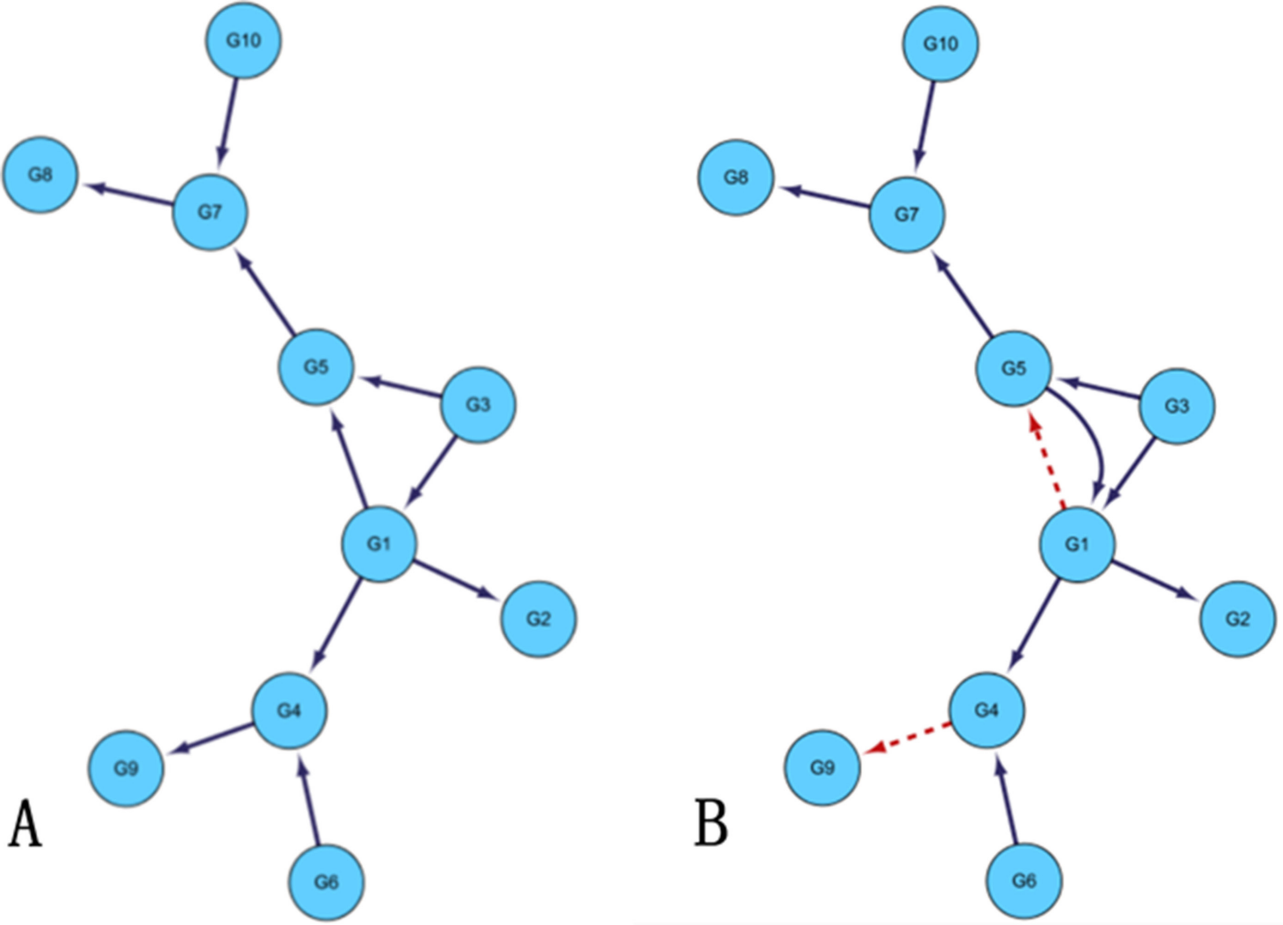
Comparison of DREAM3 10 nodes real GRNs and inferred GRNs

First, we analyze yeast gene expression data with network size 10 and sample number 10. In order to obtain different sparsity of the regulatory networks, which need to set some parameters in the algorithm. For example, the conditional independence order of the network can be restricted. Generally, the higher the order is, the higher the sparsity is. For the threshold value of determining whether the genes are correlated randomly, the bigger the threshold is, the more the network sparsity is. In the course of the experiment, the determination independence threshold is set to 0.03, without limiting the network order, that is, until there is no higher order CMI2 calculation. The final order of the network is 1st order. Set the maximum time-delayed unit *k* = 5, the weighting coefficient ɷ is 0.09. Figure 1 is the gold standard GRNs and inferred GRNs by the DBNCS algorithm. Figure 1 A is the standard network, the network contains 10 nodes with 10 edges, the network is roughly tree-like structure. The network is a small-scale network selected from the yeast genome network database and has been experimentally verified. Figure 1B shows that the inferred GRNs by the DBNCS algorithm. Figure 1 is plotted using the biological network analysis software Cytoscape (http://www.cytoscape.org/) [45, 60].

In the DREAM3 10-node network structure, DBNCS algorithm can successfully infer most real network structures, with only *G*4–*G*9 edge being missed. It can be seen from Figure 1 that the highest order mutual information existing between gene *G*4 and gene *G*9 is zero-order mutual information, but *I_G_*_4–*G*9_ = 0.0244 is less than the judgment independence threshold θ = 0.03. In addition, 8 edges out of the remaining 9 edges can be derived correctly by our model. This proves that the DBNCS algorithm can detect most of the network regulation directions without transcription factor information.

The transcriptional time-delayed between the regulation relationships calculated in the search space is calculated by the CMI2 values as shown in Table 1. It can be seen from Table 1 that the network structure (priori network) of 10-node multiple time-delayed GRNs consists of nine groups of regulation relations such as *G7→G8*, *G10→G7,* as shown in Figure 1B. The transfer network of the multi-delayed GRN is composed of the regulatory delay corresponding to the nine groups of regulation relations. For example, the gene *G*8 is regulated by *G*7 after two time units, and the gene *G*7 is regulated by *G*10 after four time units.

**Table 1:** DBNCS algorithm is used to infer the time-delayed of DREAM3 10-node GRNs

Regulation relation	Time unit	Regulation relation	Time unit
G7→G8	2	G1→G5	4
G10→G7	4	G1→G2	1
G5→G7	4	G1→G4	4
G3→G5	1	G6→G4	1
G3→G1	2		

The optimal network structure was constructed by using the comprehensive score model in the search space to construct the optimal network structure. The true positive rate (TPR), false positive rate (FPR), positive predictive value (PPV), overall accuracy (ACC), Matthew's correlation coefficient (MCC) and the area under curve (AUC) are used to evaluate the performance of each network construction algorithm. Table 2 shows the main index values of DBNCS algorithm and other reference methods to infer GRNs.

**Table 2:** Comparison of the different methods’ performances on the DREAM3 10 gene dataset

Method	TPR	FPR	PPV	ACC	MCC	AUC
GENIE3	0.700	0.112	0.437	0.867	0.483	0.919
ARACNE	0.900	0.112	0.500	0.888	0.618	0.930
NARROMI	0.700	0.050	0.636	0.922	0.623	0.938
DBNCS	0.800	**0.013**	**0.889**	**0.967**	**0.825**	**0.942**

**Figure 2:**
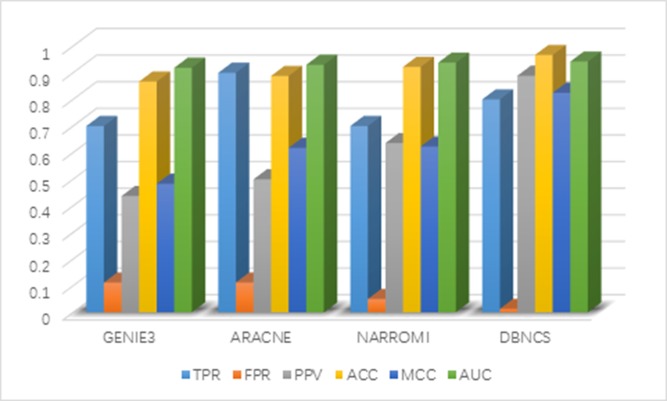
Comparison of the results of constructing DREAM3 10-node GRNs with DBNCS algorithm

It can be seen from Table 2 that the FPR, PPV, ACC, MCC and AUC five indicators of DBNCS algorithm are all the best of these algorithms, achieved 0.013, 0.889, 0.967, 0.825 and 0.942, respectively. The TPR is second only to ARACNE algorithm, reaching 0.800. Figure 2 shows the main results of the three algorithms and the DBNCS algorithm to infer GRNs.

The FPR of the DBNCS algorithm is 9.9% lower than GENIE3 and ARACNE algorithm, which is 3.7% lower than that of NARROMI algorithm. The PPV is 45.2% higher than GENIE3 algorithm and 25.3% higher than NARROMI algorithm. The ACC is 10% higher than GENIE3 algorithm and 4.5% higher than NARROMI algorithm. Matthew's correlation coefficient (MCC) is 34.2% higher than GENIE3 algorithm, and 20.2% higher than NARROMI algorithm. The AUC is 2.3% higher than GENIE3 algorithm and 0.4% higher than NARROMI algorithm.

In the comparison algorithm, GENIE3 and NARROMI have better performances in the construction of small-scale regulatory networks. However, it is difficult for ARACNE algorithm to derive higher-order networks without introducing some low-order network information. Hamiltonian truncation is also prone to serious systematic error because it cannot easily identify the interaction between pairs of genes, so although ARACNE algorithm has the highest true positive rate in the prediction results, its positive predictive value is not a very good performance. In contrast, in the construction of network structure profiles, DBNCS algorithm uses CMI2 to explore the nonlinear relationship between variables, while avoiding calculation instability caused by the special data. In addition, the DBNCS algorithm uses the comprehensive score model to fully excavate the information in the gene expression data. Compared with the scoring model used by other GRNs construction algorithms, the comprehensive score model is constructed by adding the consideration of gene transcription time delay, so that the GRN is more in line with the real network. Three aspects of information are taken into account to ensure the stability of the DBNCS algorithm.

### DREAM3 challenge 50 Nodes GRNs cnstruction

In this section, we analyze yeast gene expression data with network size 50 and sample number 50. The real network of the data is selected from the yeast genome database and is a medium-scale network. The CMI2NI algorithm is used to construct the network structure profiles through the gene expression data, that is, the search space. Set the judgment independence threshold *θ* = 0.023, which does not limit the network order, and the final order of the network is 4th order. Set the maximum time-delayed unit *k* = 5, the weighting coefficient ɷ is 0.9.

**Figure 3:**
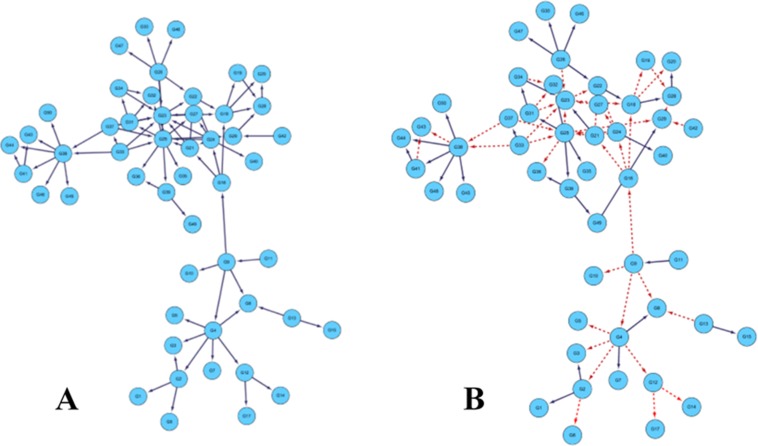
Comparison of DREAM3 50 nodes real GRNs and inferred GRNs

Figure 3 is the gold standard GRNs and inferred GRNs by the DBNCS algorithm. Figure 3A is the standard network, the network contains 50 nodes with 77 edges. Figure 3B shows the inferred GRNs by the DBNCS algorithm from the gene expression data. The inference network predicts a total of 77 edges, of which 31 are true-positive, 48 false-positive and 46 false negative. In Figure 3B, the solid line indicates the correctly inferred edges, and the broken line represents the edges which are not correctly inferred compared to the real network.

After the redundant regulate of the search space, the CMI2 values are used to calculate the transcription time delay of each regulation relationship in the search space, as shown in Table 3. From Table 3, the network structure of the multiple time-delayed GRNs inferred by the DBNCS algorithm and conforming to the real network consists of 31 groups of regulation relationships as *G2→G1*, *G*2*→G*3, and so on, as shown in Figure 3B. For example, gene *G*1 is regulated by *G*2 after the three time units, gene *G*7 is regulated by *G*4 after the five time units. Table 4 shows the main indicators of the DBNCS algorithm and other methods to infer GRNs.

**Table 3:** DBNCS algorithm is used to infer the time-delayed of DREAM3 50-node GRNs

Regulation relation	Time unit	Regulation relation	Time unit	Regulation relation	Time unit
G2→G1	3	G25→G32	2	G38→G41	1
G2→G3	3	G25→G35	3	G38→G44	1
G4→G7	5	G25→G27	3	G38→G45	5
G4→G8	2	G26→G22	1	G38→G48	2
G11→G9	1	G26→G30	4	G38→G50	2
G13→G15	1	G26→G46	3	G39→G36	5
G18→G28	4	G26→G47	1	G39→G49	2
G21→G23	5	G28→G20	1	G41→G44	1
G22→G18	3	G31→G34	5		
G24→G40	1	G33→G37	3		
G25→G31	3	G34→G23	3		

**Table 4:** Comparison of the different methods’ performances on the DREAM3 50 gene dataset

Method	TPR	FPR	PPV	ACC	MCC	AUC
GENIE3	0.481	0.078	0.167	0.908	0.245	0.843
ARACNE	0.597	0.082	0.192	0.908	0.303	0.832
NARROMI	0.532	0.062	0.217	0.925	0.307	0.839
DBNCS	0.416	**0.019**	**0.416**	**0.963**	**0.397**	**0.848**

**Figure 4:**
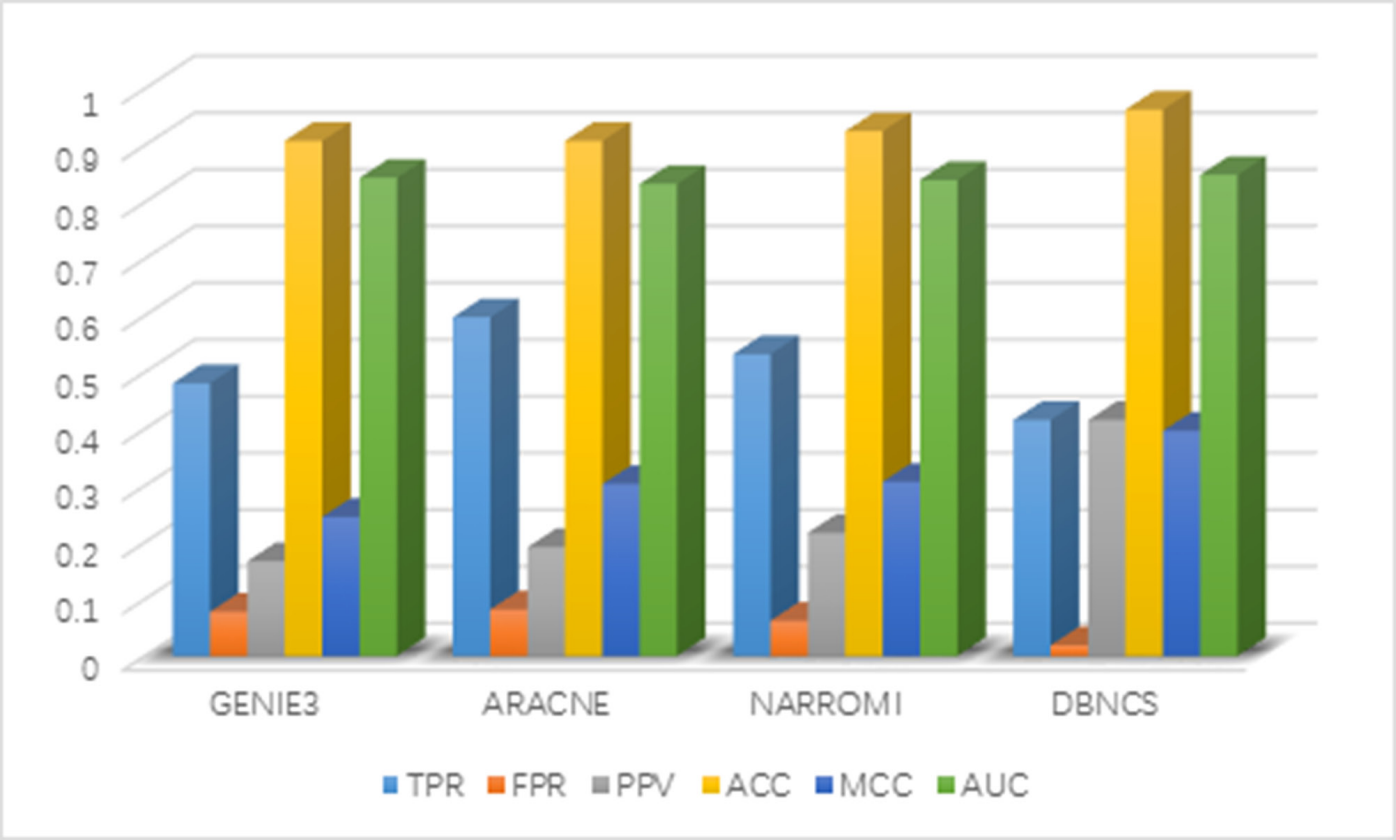
Comparison of the results of constructing DREAM3 50-node GRNs with DBNCS algorithm

It can be seen from Table 4 that the FPR, PPV, ACC, MCC and AUC five indicators of DBNCS algorithm are all the best of these algorithms, achieved 0.019, 0.416, 0.963, 0.397 and 0.848, respectively. The true positive rate (TPR) is 0.416. Figure 4 shows the main results of the three algorithms and the DBNCS algorithm to infer GRNs.

For the construction of medium-sized network, DBNCS algorithm as a whole is significantly better than other three algorithms. The TPR of DBNCS algorithm is 0.416, which is 18.1% lower than ARACNE algorithm, and 7.8% lower than GENIE3 algorithm. The FPR is 6.3% lower than ARACNE algorithm and 4.2% lower than NARROMI algorithm. DBNCS is 24.9% higher than GENIE3 in terms of PPV and 17.5% higher than the NARROMI algorithm. The ACC is 5.5% higher than GENIE3 algorithm and 3.7% higher than NARROMI algorithm. Matthew's correlation coefficient (MCC) is 15.2% higher than GENIE3 algorithm, and 7.1% higher than NARROMI algorithm. The AUC is 0.5% higher than GENIE3 algorithm and 7.1% higher than NARROMI algorithm.

Although the ARACNE algorithm has the highest TPR, other indicators are not ideal. GENIE3 algorithm from the gene expression data mining information is single, very sensitive to the parameter changes, the overall stability is poor, under the conditions of fixed parameters, the results of each operation are not the same, and occasionally there will be lower accuracy rate. The comprehensive score model used in the DBNCS algorithm fully excavates the linear correlation and non-linear correlation of the gene expression data. Compared with other algorithms, the comprehensive score model is added to the consideration of the dynamic characteristics of genes. The construction of the regulatory network is more in line with the biological basic physiological mechanism, which realizes the accurate measurement of the causal intensity between genes. On the basis of determining the search space, the false positive rate of the network construction is reduced, and effectively avoids the structural loss caused by the single model feature, which ensures the stability of the algorithm.

### Network construction of real data of E. coli SOS DNA repair network

**Figure 5:**
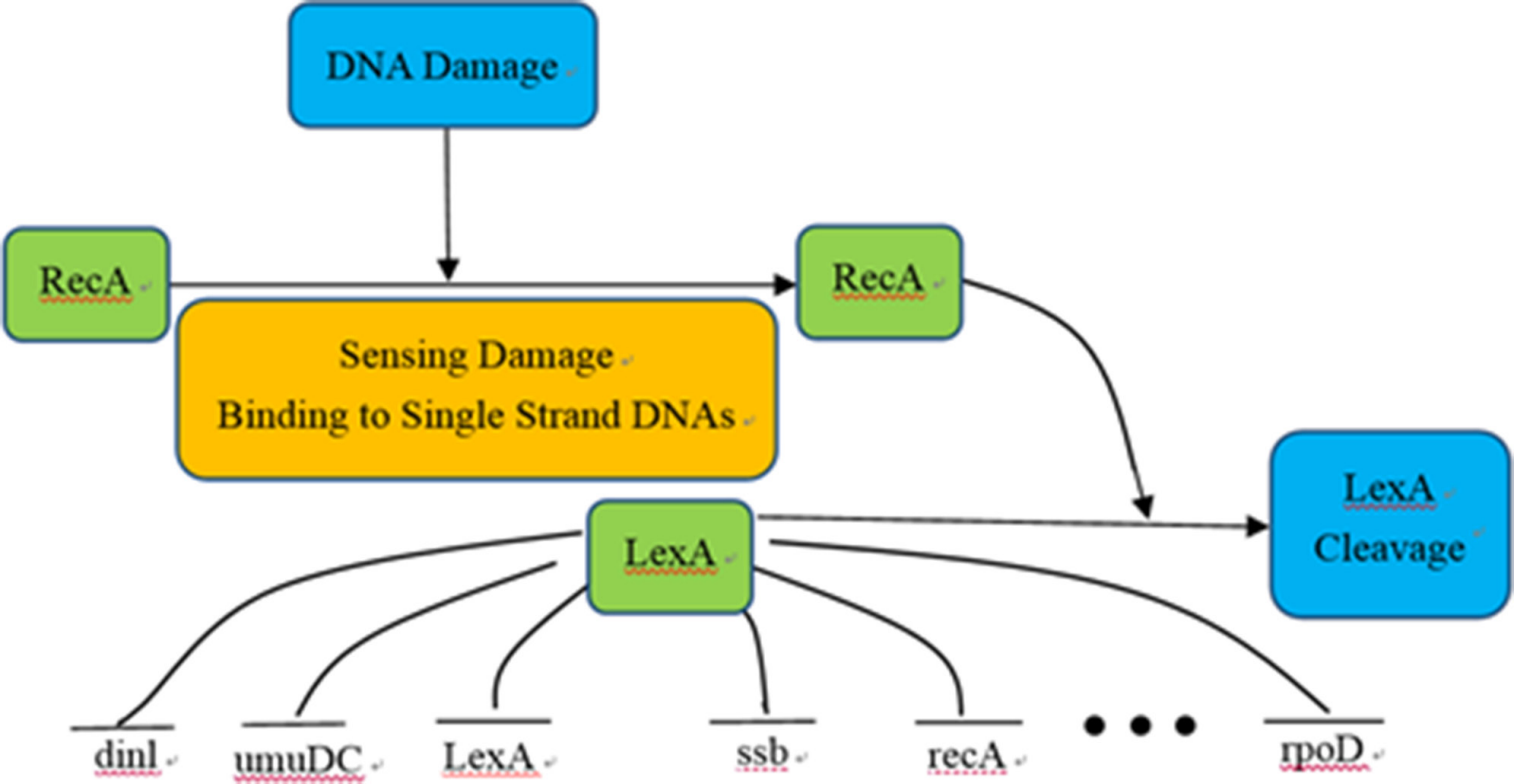
SOS DNA repair system of *Escherichia coli*

To investigate the performance of the DBNCS algorithm to construct GRNs through real gene expression data, we use the signaling pathways of the *E. coli* SOS DNA repair system to perform network reconstruction, as shown in Figure 5. The network is often used to verify the effectiveness of the algorithm [61–66].

The network is an *E. coli* SOS DNA repair system, which contains 9 genes. The main function of the SOS signaling pathway is to regulate cellular immunity and repair after DNA damage, including the key genes LexA and recA, as well as their directly regulated more than 30 genes and dozens or even hundreds of indirectly regulated genes [63].

**Figure 6:**
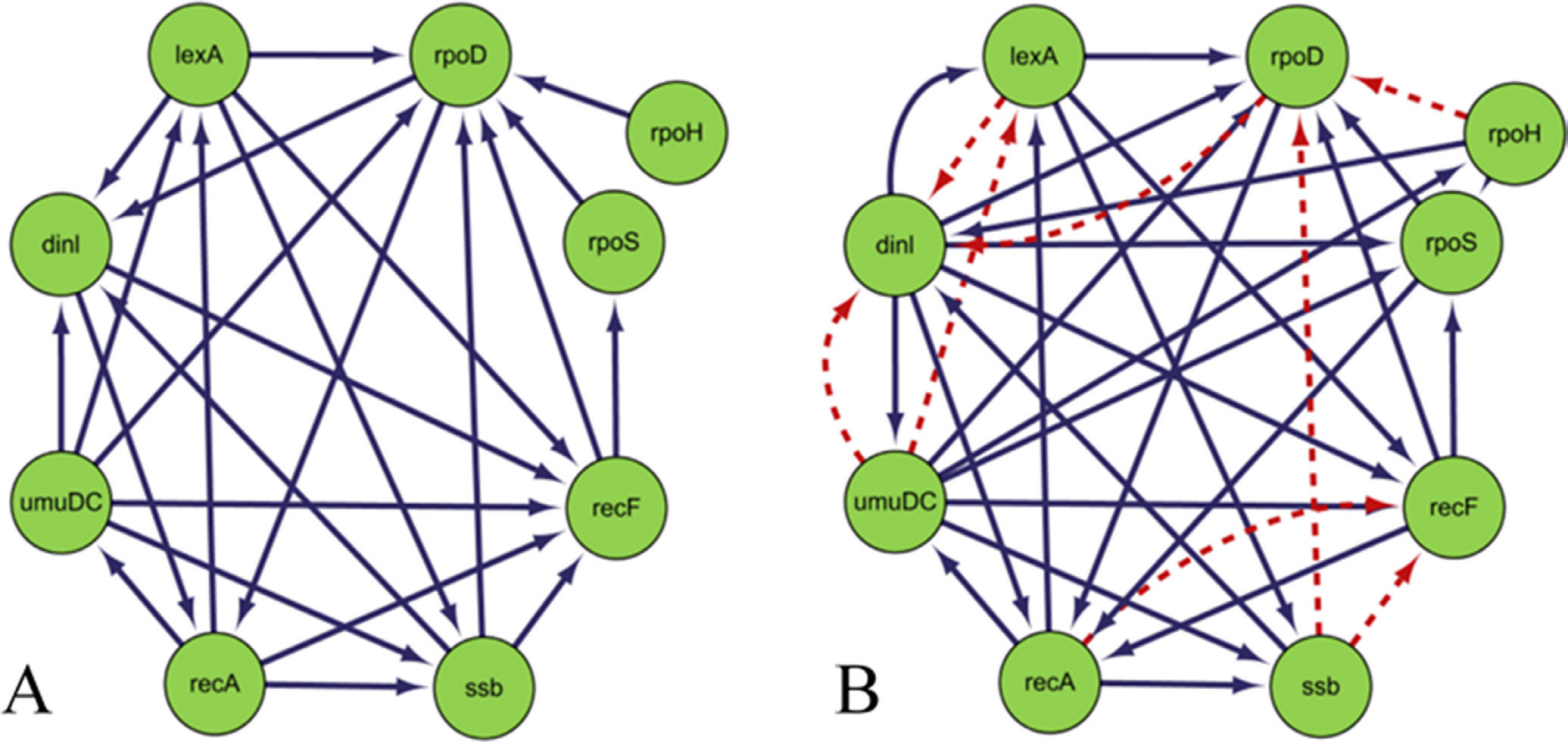
Comparison of *Escherichia coli* SOS DNA repair network and inferred gene regulatory network

Construction SOS DNA repair network in *E. coli* is similar to that of the DREAM3 challenge yeast GRN, here we only construct static GRN. In this paper, the CMI2NI algorithm is used to study the network structure profiles, and the search space is constructed. The judgment independence threshold is 0.001, which does not limit the network order, and the final order of the network structure is 7th order. The weighting coefficient of the CMI2 value and the RO is ɷ = 0.3. After the redundant regulations are removed, the optimal network structure is constructed by using the comprehensive score model in the search space to carry out the greedy search. (The trade-off between the *β* value and the TRS value is σ = 1, so that the comprehensive score model only considers the linear and non-linear components in the gene expression data when calculating the score, thus ignoring the mining of the dynamic feature information.) The TPR, FPR, PPV, ACC and Matthew's correlation coefficient are used to evaluate the performance of the algorithm. The inferred results are shown in Table 5. Figure 6 is the gold standard GRNs and inferred GRNs by the DBNCS algorithm. Figure 6A shows the real SOS DNA repair network in *E. coli*, which contains 9 nodes and 24 edges. Figure 6B shows the referred GRNs by DBNCS algorithm from the gene expression data. The solid line indicates the correctly inferred edges, and the broken line represents the edges which are not correctly inferred compared to the real network. In the inference network, a total of 27 edges are predicted, of which 16 are true-positive, 11 are false-positive and 37 are false-negative.

**Table 5:** Comparison of the different methods’ performances on the SOS DNA repair network

Method	TPR	FPR	PPV	ACC	MCC	AUC
GENIE3	0.500	0.208	0.546	0.694	0.299	0.684
ARACNE	0.708	0.625	0.362	0.486	0.083	0.739
NARROMI	0.667	0.458	0.421	0.583	0.197	0.791
Grow-shring	0.458	0.271	0.458	0.639	0.188	0.758
IAMB	0.583	0.229	0.560	0.708	0.351	0.809
DBNCS	0.667	0.229	**0.593**	**0.736**	**0.426**	**0.813**

**Figure 7:**
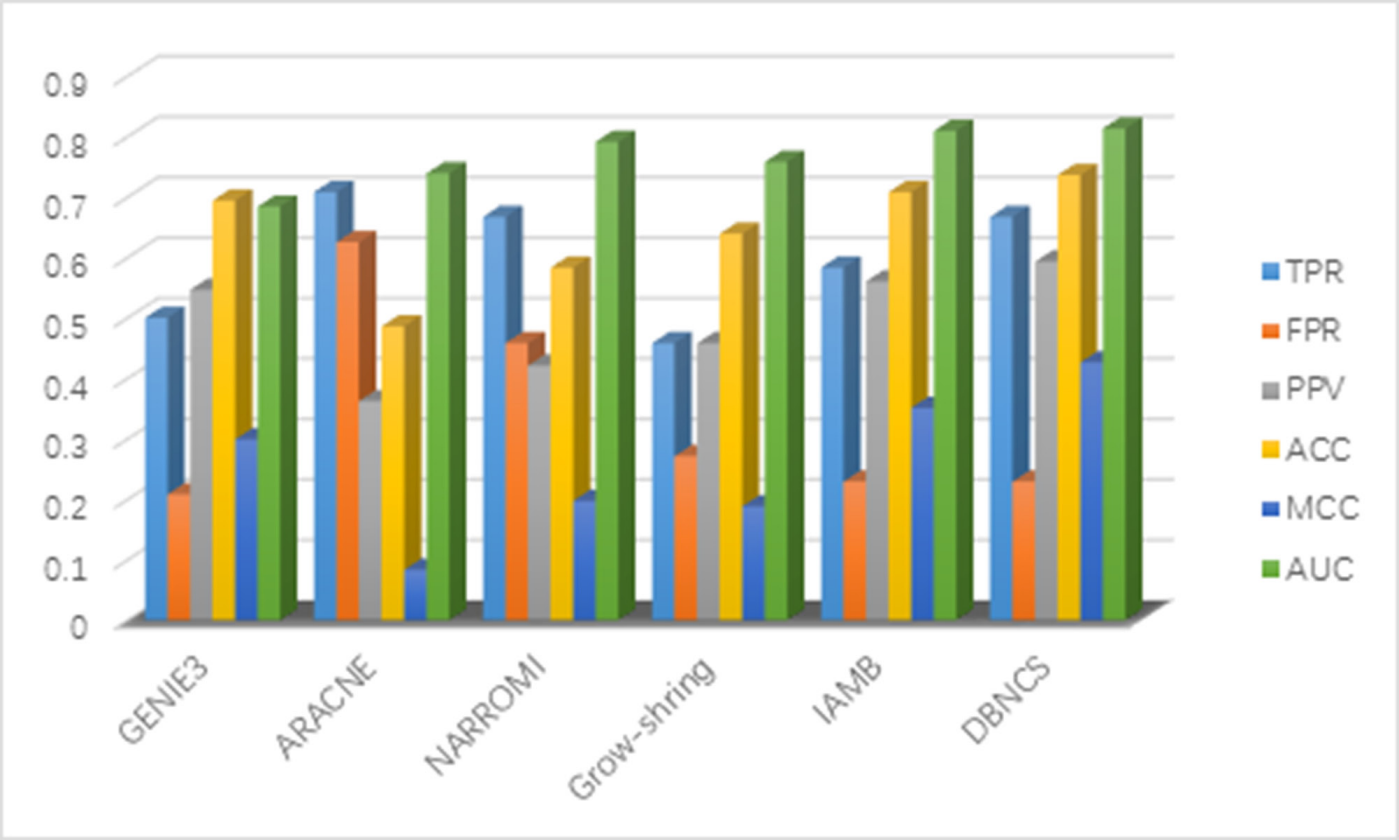
Comparison of the results of constructing SOS DNA repair network in *E. coli* 9-node GRNs with DBNCS algorithm

It can be seen from Table 5 that the PPV, ACC, MCC and AUC four indicators of DBNCS algorithm are all the best of these six algorithms, which are 0.593, 0.736, 0.426 and 0.813, respectively. The TPR and FPR are second to ARACNE and GENIE3 algorithm results, respectively, achieve 0.667 and 0.229, respectively. For the construction of SOS DNA repair network in *E. coli*, the TPR of DBNCS algorithm is 4.1% lower than ARACNE and 20.9% higher than Grow-shring algorithm. The FPR is 2.1% higher than that of GENIE3 algorithm, 39.6% lower than that of ARACNE algorithm. The PPV is 4.7% higher than GENIE3 algorithm and 23.1% higher than ARACNE algorithm. The ACC is 2.8% higher than IAMB algorithm, 25% higher than ARACNE algorithm. MCC is 7.5% higher than IAMB algorithm, and 34.3% higher than ARACNE algorithm. The AUC is 0.4%, 12.9% higher than that of IAMB and GENIE3 algorithm, respectively. Figure 7 shows the main results of the five comparison algorithms and the DBNCS algorithm to infer GRNs. On the whole, the main results of DBNCS algorithm are better than other comparison algorithms.

Compared with the lower true positive rate inferred by the GENIE3 and Grow-shring algorithm, and the higher false positive rate inferred by the ARACNE algorithm. DBNCS algorithm uses the stepwise optimization algorithm to recursively optimize the network structure profiles to eliminate the initial redundancy, and carry out optimizing repeatedly on the basis of the first optimizing, that is redundancy regulation to reduce the false positive rate of the network inference, and improve the adaptability of the DBNCS algorithm. In addition, due to the use of the comprehensive score model, that is, full mining of the linear correlation, nonlinear correlation and dynamic characteristics, DBNCS algorithm has more excellent performance than the other algorithms in the real regulatory network construction.

## MATERIALS AND METHODS

### Datasets and evaluation metrics

In order to validate our method, DBNCS was evaluated using two simulation datasets and a real gene expression dataset. The two synthetic datasets in sizes 10 and 50 obtained from DREAM3 challenge [53]. The real gene expression dataset is the well-known SOS DNA repair network with experiment dataset in *E. coli.* [34, 35], which includes 9 genes.

The performance of the proposed method were evaluated by following measures, i,e. true positive rate (TPR), false positive rate (FPR), positive predictive value (PPV), overall accuracy (ACC) and Matthew's correlation coefficient (MCC). These metrics are defined as follows:

$$\begin{array}{l} \mathrm{TPR}=\mathrm{TP}/(\mathrm{TP}+\mathrm{FN}),\;\mathrm{FPR}=\mathrm{FP}/(\mathrm{FP}+\mathrm{TN})\\ \mathrm{PPV}=\mathrm{TP}/(\mathrm{TP}+\mathrm{FP}),\;\mathrm{ACC}=(\mathrm{TP}+\mathrm{TN})/(\mathrm{TP}+\mathrm{FP}+\mathrm{TN}+\mathrm{FN})\\ \mathrm{MCC}=\frac{\mathrm{TP}\times \mathrm{TN}-\mathrm{FP}\times \mathrm{FN}}{\sqrt{\left(\mathrm{TP}+\mathrm{FP})(\mathrm{TP}+\mathrm{FN})(\mathrm{TN}+\mathrm{FP})(\mathrm{TN}+\mathrm{FN}\right)}} \end{array}$$ (1)

where TP, FP, TN and FN are the numbers of true positives, false positives, true negatives and false negatives, respectively. TPR and FPR are also used to plot the receiver operating characteristic (ROC) curves. The area under ROC curve (AUC) is calculated as another meric for comparing different algorithms.

To evaluate the performance of our method, the DBNCS algorithm was compared with the algorithm with better performance. For the DREAM3 datasets, three algorithms were selected for deriving GRNs, such as ARACNE algorithm based on mutual information [62], GENIE3 algorithm based on random forest [67] and NARROMI algorithm based on recursive optimization algorithm. For all the methods in the comparison, the parameter is set to the algorithm default parameters for calculation. For example, the parameters of the NARROMI algorithm [68] was set to 1 and the ensemble parameter of the GENIE3 method was set to 1000. For the SOS DNA repair network in *E. coli*, we selected the algorithms of ARACNE, GENIE3, NARROMI, IAMB based on hill climbing algorithm [69] and Grow-shring algorithm based on Markov blanket [70].

### Algorithm of network structure profiles construction based on conditional mutual inclusive information

#### Conditional mutual inclusive information

In recent years, mutual information (MI) and conditional mutual information (CMI) have been widely used to reconstruct GRNs [24, 36, 42, 44, 60, 71, 72], due to their capability of characterizing nonlinear dependency, which provides a natural generalization of association between genes. MI can be used to measure the dependency between two variables (genes) *X* and *Y*. On the other hand, CMI measures the conditional dependency between two variables (genes) *X* and *Y* given other variable (gene) *Z*, which can quantify the undirected regulation.

For discrete variables *X* and *Y*, MI is defined as [36, 42, 73, 74]

$$\begin{array}{lll} \mathrm{MI}(X,Y) & = & -\sum_{x\in X,y\in Y}p(x,y)\log\frac{p(x,y)}{p(x)p(y)}\\ & = & H(X)+H(Y)-H(X,Y) \end{array}$$ (2)

where *p*(*x*, *y*) is the joint probability distribution of *X* and *Y*, and *p*(*x)* and *p*(*x)* are the marginal probability distributions of *X* and *Y*, respectively. *H*(*x*) and *H*(*x*) are the entropies of *X* and *Y* and, respectively, and *H*(*x*) is joint entropy of *X* and *Y* and.

With the widely adopted hypothesis of Gaussian distribution for gene expression data, formula (2) can be easily calculated by using the following equivalent formula [36, 42, 73]

$$\mathrm{MI}(X,Y)=\frac{1}{2}\log\frac{\left|C(X)\right|\left|C(Y)\right|}{\left|C(X,Y)\right|}$$ (3)

Where *C* is the covariance matrix of variables, |C| is the determinant of the matrix *C*. If variables (genes) *X* and *Y* andare independent of each other, clearly *MI(X,Y) = 0*.

The CMI of variables *X* and *Y* given variable *Z* is defined as [36, 42, 73]

$$\begin{array}{lll} \mathrm{CMI}(X,Y|Z) & = & \sum_{x\in X,y\in Y,z\in Z}p(x,y,z)\log\;\frac{p(x,y|z)}{p(x|z)p(y|z)}\\ & = & H(X,Z)+H(Y,Z)-H(Z)-H(X,Y,Z) \end{array}$$ (4)

where *p*(*x*,*y*|*z*), *p*(*x*|*z*) and *p*(*y*|*z*) are conditional probability distributions, *H*(*X*,*Z*), *H*(*Y*,*Z*) and *H*(*X*,*Y*,*Z*) are joint entropies.

Similarly, formula (4) can be easily calculated by using the following equivalent formula [36, 42]

$$\mathrm{CMI}(X,Y|Z)=\frac{1}{2}\log\frac{\left|C(X,Z)\right|\left|C(Y,Z)\right|}{\left|C(Z)||C(X,Y,Z)\right|}$$ (5)

Obviously, if the variables *X* and *Y* are conditional independence given *Z, CMI*(X,Y|Z)=0. In addition, fomula (5) is an efficient method to calculate CMI between two variables (genes) *X* and *Y* given one or more variables *Z*. For example, if the conditional variable Z = (*Z*_1,_*Z*_2_) is composed of two variables (genes) Z_1_ and Z_2_, we can obtain the second-order CMI.

MI tends to overestimate the regulation strengths between genes (i.e., false positive problem), while CMI tends to underestimate the regulation strengths (i.e., false negative problem). In order to overcome these problems, a new concept CMI2 (conditional mutual inclusive information) quantify causal strength between genes [71].

In a directed acyclic graph (DAG), if variable *Y* is regulated by variable *X* both directly and indirectly through variable *Z*, CMI2 is defined as

$$\begin{array}{l} \mathrm{CMI}2(X;Y|Z)\;=\\ \mathrm{CMI}(X;Y|Z)+\frac{1}{2}D_{\mathrm{KL}}(P(Y|Z)\|P_{X\to Y}(Y|Z))+\frac{1}{2}D_{\mathrm{KL}}(P(X|Z)\|P_{Y\to X}(X|Z)) \end{array}$$ (6)

The above formula can be decomposed into three terms, one of them is CMI, and another two are non-negative Kullback-Leibler (KL) divergence. Where *P_X→Y_* and *P_Y→X_* are the interventional probability distribution of *X*,*Y and Z* for removing edges _*X→Y*_ and _*Y→X*_, respectively. _*DKL*(_*P||P_X→Y_*) and _*DKL*(_*P||P_Y→X_*) are the KL divergences from *P* to _*PX→Y*_ and _*PY→X*_, respectively.

According to the definition of KL-divergence and conditional mutual inclusive information, *CMI*_2(_*X*_;_*Y*_|_*Z*) can be rewritten as

$$\mathrm{CMI}2(X;Y|Z)=\;\sum_{x,y,z}\;P(x,y,z)\ln\frac{P(x,y,z)}{P(x,z)\sum_{x}P(y|z,x)P(x)+P(y,z)\sum_{y}P(x|z,y)P(y)}$$ (7)

where *P(y|z,x)* and *P(x|z,y)* are the conditional probabilities.

In a DAG, if variables *X* and *Y* be 1-dimension, variable *Z* is a *n_z_*-dimension (*n*_z ≥ 1)_, and *X,Y* and Z, obey Gaussian distribution. Then

$$\mathrm{CMI}2(X;Y|Z)=\frac{1}{4}(\mathrm{tr}(C^{-1}\Sigma )+\mathrm{tr}({\tilde{C}}^{-1}\tilde{\Sigma })+\ln C_{0}+\ln{\tilde{C}}_{0}-2n)$$ (8)

where,

$$C_{0}=\rho_{xx}({\left(\Sigma^{-1}\right)}_{xx}-{\left(\Sigma_{1}^{-1}\right)}_{xx}+\rho_{xx}^{-1})$$

$${\tilde{C}}_{0}=\rho_{yy}({\left({\tilde{\Sigma }}^{-1}\right)}_{yy}-{\left({\tilde{\Sigma }}_{1}^{-1}\right)}_{yy}+\rho_{yy}^{-1}),\;n=n_{z}+2\text{,}$$

$$C={\left(\begin{array}{ccc} C_{xx} & C_{xy} & C_{xz}\\ C_{xy} & C_{yy} & C_{yz}\\ C_{xz}^{T} & C_{yz}^{T} & C_{zz} \end{array}\right)}^{-1}\text{,}$$

$$\tilde{C}={\left(\begin{array}{ccc} {\tilde{C}}_{yy} & {\tilde{C}}_{yx} & {\tilde{C}}_{yz}\\ {\tilde{C}}_{yx} & {\tilde{C}}_{xx} & {\tilde{C}}_{xz}\\ {\tilde{C}}_{yz}^{T} & {\tilde{C}}_{xz}^{T} & {\tilde{C}}_{zz} \end{array}\right)}^{-1}\;\Sigma =\left(\begin{array}{ccc} \rho_{xx} & \rho_{xy} & \rho_{xz}\\ \rho_{xy} & \rho_{yy} & \rho_{yz}\\ \rho_{xz} & \rho_{yz} & \rho_{zz} \end{array}\right)\text{,}$$

$$\tilde{\Sigma }=\left(\begin{array}{ccc} \rho_{yy} & \rho_{yx} & \rho_{yz}\\ \rho_{yx} & \rho_{xx} & \rho_{xz}\\ \rho_{yz} & \rho_{xz} & \rho_{zz} \end{array}\right)\text{, }\Sigma_{1}=\left(\begin{array}{cc} \rho_{xx} & \rho_{xz}\\ \rho_{xz} & \rho_{zz} \end{array}\right)\text{,}$$

$${\tilde{\Sigma }}_{1}=\left(\begin{array}{cc} \rho_{yy} & \rho_{yz}\\ \rho_{yz} & \rho_{zz} \end{array}\right)\text{.}$$

where *ρ* is the Pearson correlation coefficients of random variables; $\Sigma_{1}$, ${\tilde{\Sigma }}_{1}$ are the covariance matrices of variables (genes) *x*,*z* and *y*,*z*, respectively; $C,\;\tilde{C}$, Σ, $\tilde{\Sigma }$ are the covariance matrices of variables (genes) *x*,*y* and *z*.

Under the assumption of Gaussian distribution of gene expression data, the CMI2 can be easily calculated by formula (8) using the covariance coefficients and the Pearson correlation coefficients of the gene expression data.

### CMI2NI algorithm [71]

CMI2NI algorithm is GRN construction algorithm developed by combining CMI2 algorithm and path consistency algorithm (PCA) [44]. First, a complete graph is constructed according to the number of genes. Second, calculate the MI of each pair of adjacent genes. If the MI value between genes is less than a given threshold, then there is no regulatory relationship between the pair of genes and delete the edges connecting the two points in the complete graph. Subsequently, for those pairs of genes in the treated graph which still have the regulatory relationship, *k* genes are selected from the genome which are connected to these two genes as a conditional independent judgment condition gene, calculate *k* th-order CMI2, determine independence between the pair of genes, and delete the edge which connected the two genes to update the network structure profiles. The next step continues to compute the higher order CMI2 and gradually remove redundant edges in a network based on the conditional independency between the nodes, until no higher order CMI2 can be calculated. The resulting sparse network will be used as the final network structure profiles, namely the search space.

In order to reduce the computational complexity without reducing the accuracy of the network inference, the CMI2NI algorithm adopts an optimal strategy to select the subset of genes from the sets that is adjacent to the pair of genes to be judged as the conditional gene. For example, suppose that there are *T*(*T*≥1) genes which are adjacent with both genes *i* and *j*, when constructing *L* th-order (*L*≤*T*) network, all the *L*th-order CMIs for the possible combinations of *L* conditional genes from *T*genes are computed and the maximum or the geometric mean of them is selected to determine the existence of the regulatory relationship. Generally, after a few number of iterations *L*, the computation will terminate due to no further change in the network topology.

### The network structure comprehensive score model based on recursive optimization algorithm (RO), MI and transcriptional regulation score (TRS)

According to the theory of Jensen et al. [72] and Mengshoel et al. [75, 76], the Bayesian network can be divided into several cliques without loss. The clique's internal vector must be connected in pairs, and the smallest clique consists of two vectors. Based on the profiles of the network structure, the genes with non-random regulatory relations are regarded as cliques and the optimal network structures are searched to get the optimal multiple time-delayed GRNs. The scoring model is an important guarantee for the construction of network structure with high precision in the process of searching for the optimal network structure. Several scoring models, such as Bayesian metric, minimum description length metric, Bayesian information criterion and other model structures are relatively single. In this study, a new network structure comprehensive score model is proposed and applied to a DBN to achieve a comprehensive scoring of the DBN structure. In addition, in this paper, the recursive optimization algorithm, which is used to construct the GRN, is used to remove the redundant regulations of the network structure profiles, and the calculated regulation strength vector is applied to the comprehensive score model through the nonlinear combination.

### Recursive optimization

For a target gene with expression level *y*, define a regulation intensity vector *β* to fit the expression matrix *X* of the transcription factors. *β* can be estimated by minimizing the error between the observed value and the inferred value, i.e.

$$\underset{\beta }{\min}|y-\beta X|+\lambda |\beta |$$ (9)

where *X* is the expression matrix of the transcription factor, λ is a non-negative parameter used to balance the sparsity and error term in the objective function.

In general, the activity of a transcription factor is proportional to the gene expression level encoding the corresponding transcription factor, so that the gene expression level can be used instead of the activity of the transcription factor, thus the model (9) is equivalent to

$$\underset{\beta^{j}}{\min}\sum_{i=1}^{m}\left|y^{i}-\sum_{j=1}^{c}\beta^{{}^{j}}x_{j}^{i}\right|+\lambda \sum_{j=1}^{c}\left|\beta^{{}^{j}}\right|$$ (10)

Let

ui+vi=|yi−∑j=1cβjxji| , ui−vi=yi−∑j=1cβjxji,ξj≡ηj |βj| , ξj-ηj βj, i=1,2,⋯,m, j=1,2,⋯,c

where $u_{i},v_{i},\xi_{j},\eta_{j}\geq 0$ then model (10) can be written as a linear programming (LP) model as follows.

$$\begin{array}{l} \underset{u_{i},v_{i},\xi_{j},\eta_{j}}{\min}\sum_{i=1}^{m}(u_{i}+v_{i})+\lambda \sum_{j=1}^{c}\left(\xi_{j}+\eta_{j}\right)\\ \;s.t.\;u_{i}-v_{i}=y^{i}-\sum_{j=1}^{c}(\xi_{j}-\eta_{j})x_{j}^{i}\\ u_{i},v_{i},\xi_{j},\eta_{j}\geq 0. \end{array}$$ (11)

The above LP model (11) can be solved efficiently by any LP software such as GLPK LP/MIP solver [77]. Although the parameters λ in model (11) can control the network sparseness to a certain extent, it is difficult to obtain the optimal solution of the network structure even in the cliques because of the noise in the gene expression data [78–80]. In order to solve this problem without reducing the false positives, a new threshold *θ*_0_ (if the clique is of the simplest form of the gene pair, then *θ*_0 =_ *θ*) is set and the low regulation strength of the gene pair is set to zero, and the non-zero variables in the model (10) are re-estimated in the next step. Through this reoptimization solution, the influence of noise and redundancy regulation can be removed to a certain extent, and improve the accuracy of the GRN inference with clique as the units. To maximize accuracy, the above procedure will be repeated until there are no non-zero variables that need to be re-estimated [68].

### Transcriptional delay calculation and transcriptional regulation score

The gene expression profile of the target gene relative to its regulatory gene delayed a different unit of time. The larger of MI value calculated by the two genes, the greater the probability that the two genes will have a regulation relationship under the delay. The MI algorithm can be used to derive the transcriptional delay between genes with regulatory relations. First of all, given the maximum time delay unit allowed in the calculation *k*(*k*=1,2,…,*T*–1), we set the delay time unit *k* corresponding to the existence *k* order Markov assumption, *T* represents the maximum time point of the time series gene expression data. We assume gene expression profile of gene *Y* is *Y*=(*y*[1],…y[T]), the regulated target gene is *X=(x*[1],…*x*[T]). Assuming that the time delay between *X* and *Y* is *m*(*m*=1,2,..*k*), *X*^(*m*)^=(*x*[1],…*x*[*T*–*m*]) and *Y*^(*m*)^=(*y*[m+1],…*y*[*T*]), *MI*(*X*^(*m*)^, Y^(m)^) is calculated. The time delay *m* in which the MI value is maximized is recorded as the transcription delay between *X* and *Y*. It should be noted that in the absence of knowing the direction of regulation, it is necessary to calculate the transcription delay *X* versus *Y* and *Y* versus *X*, respectively. And thus we can get a transcription time delay matrix *N*×*N*, *N* represents the number of genes.

On the basis of the gene transcription time delay, a novel transcriptional regulation score (TRS) was proposed. The TRS method was used to evaluate the network transcription score by calculating the data after recombination. The TRS evaluation reflects the true nature of gene regulation: a gene *X* may be a regulatory gene of *Y*, only if the initial change in the gene expression value *X* precedes the initial point of change in gene expression *Y*. The formula of TRS as follows

$$\mathrm{TRS}=1-\frac{1}{N_{\mathrm{IC}}}\sum_{t=0}^{T-1}\{\prod_{i=1}^{N_{v}}f(x_{t+1}^{i}-x_{t}^{i})\}\cdot \frac{\left(y_{t+2}-y_{t+1}\right)}{\left|y_{t+2}-y_{t+1}\right|}$$ (12)

where, $f(k)=\left\{ \begin{array}{l} 1\;,\;k\neq 0\\ 0\;,\;\mathrm{else} \end{array}\right.$, *N_IC_* is the total number of time points for the regulation of gene expression changes, *T* is the time points, *N_v_* is the number of regulating genes.

In the calculation of TRS values between pairs of genes, the calculation data need to be used to recombine the original data based on the determined results of the transcriptional delay. The TRS value obtained on the basis of this data reorganization is in accordance with the requirements of multiple time-delayed GRN construction and the obtained TRS score is used as the network transcription evaluation score.

### Comprehensive score model

In the process of constructing the GRN using DBN model, the scoring model plays a decisive role in identifying the direction of regulation between genes. In this paper, a new scoring model of network structure, comprehensive score (CS), is proposed in order to accurately calculate the causal intensity of genes and improve the accuracy of network construction. In this method, the linear correlation, non-linear correlation and dynamic characteristics of gene expression data are mined. In particular, the consideration of the dynamic characteristics of genes makes the construction of GRNs more in line with the biological basic physiological mechanism and closer to the real network [81–84]. The full mining of the above information allows the method to accurately calculate the causal intensity between genes, effectively reduce the false positive rate of network construction, to avoid the structure caused by a single model missing, for example the linear model of data processing will lose the gene expression data nonlinear part of the model, the nonlinear model will lose part of the linear model [61].

The numerical method of CS as follows

$$\mathrm{CS}=\sqrt{\sigma \cdot {\left(\omega |\beta^{RO}|+(1-\omega )\beta^{MI}\right)}^{2}+(1-\sigma )\cdot {\left(\mathrm{TRS}\right)}^{2}}$$ (13)

where *β^RO^* is the regulation intensity obtained by the RO algorithm, which is non-zero number; parameter ɷ is the weighting coefficient for MI and RO, and the range is [0, 1]. *β^MI^* is the calculation value by MI, which is a non-negative real number. TRS is transcriptional time-delay score calculated through TRS model. *β^RO^*, *β^MI^* and TRS value involved in calculation are all normalized values, the standardization process is achieved by background standardization. Parameter σ is a trade-off between *β* and TRS value and the range is [0, 1] (default: σ=0.6).

It is worth noting that the use of TRS method requires time-series gene expression data, and the calculation of *β* value when there is no such requirement, that is, the data required for the two numerical calculations can be different. It is because in order to obtain a multi-delay dynamic evaluation function values must use time-series gene expression data, and determining the direction of regulation does not have this need. The GRN structure information in the time-series gene expression data and the non-time-series gene expression data are included. Standardization of the three parts of the numerical values can eliminate the differences caused by different data.

### Dynamic Bayesian network

Dynamic Bayesian network (DBN) is an extension of Bayesian network that is able to infer the interaction uncertainties among genes by using a probabilistic graphical model [85]. DBN introduces the time concept and models a stochastic temporal process of a set of random variables over time series [86]. It has been employed to describe the qualitative nature of the dependencies that exist between genes in a temporal process.

A DBN is defined as a pair $\left(B_{0},\;B_{\to }\right)$ representing the joint probability distribution over all possible time series of variables $X=(X_{1},X_{2},\cdots ,X_{n})$, where $X_{i}\left(i=1,2,\cdots ,n\right)$ represents the *i* th node in the Bayesian network. In addition, the lowercase $x_{i}\left(i=1,2,\cdots ,n\right)$ represents the values of variable *X*_i_. It is composed of initial states of a Bayesian network $B_{0}=(G_{0},\Theta_{0})$ and a transition Bayesian network $B_{\to }=(G_{1},\Theta_{1})$, where *B*_0_ specifies the joint distribution of the variables in *X*[0] and $B_{\to }$ specifies the transition probabilities Pr(X[t+1]|X[t]) for all time *t*. In slice 0, the parents of $X_{i}[0]$ are assumed to be those specified in the prior network *B*_0,_ which means $\mathrm{Pa}(X_{i}[0])\subseteq X[0]$ for all 1≤i≤n; in slice t+1, the parents of $X_{i}[t+1]$ are nodes in slices *t*, $\mathrm{Pa}(X_{i}[t+1])\subseteq X[t]$ for all 1≤i≤n; the connections only exist between consecutive slices. The joint probability distribution over a finite list of random variables X[0],X[1],X[2],⋯,X[T] can be expressed as [15, 16]

$$\begin{array}{l} \mathrm{Pr}(X[0],X[1],\cdots ,X[T])=\mathrm{Pr}(X[0])\overset{T-1}{\underset{t=0}{\Pi }}\mathrm{Pr}(X[t+1]|X[t])\\ \;=\overset{n}{\underset{i=1}{\Pi }}\mathrm{Pr}(X_{i}[0]|\mathrm{Pa}(X_{i}[0]))\times \overset{T-1}{\underset{t=0}{\Pi }}\overset{n}{\underset{i=1}{\Pi }}\mathrm{Pr}(X_{i}[t+1]|\mathrm{Pa}(X_{i}[t+1])) \end{array}$$ (14)

DBN model can explore the general network structure of gene regulations and overcome the shortcomings of the acyclic assumption and static network structure in Bayesian network model. A more complicated time-varying DBN model of describing the time-evolving network structures underlying the time series is also developed.

### DBNCS algorithm

**Figure 8:**
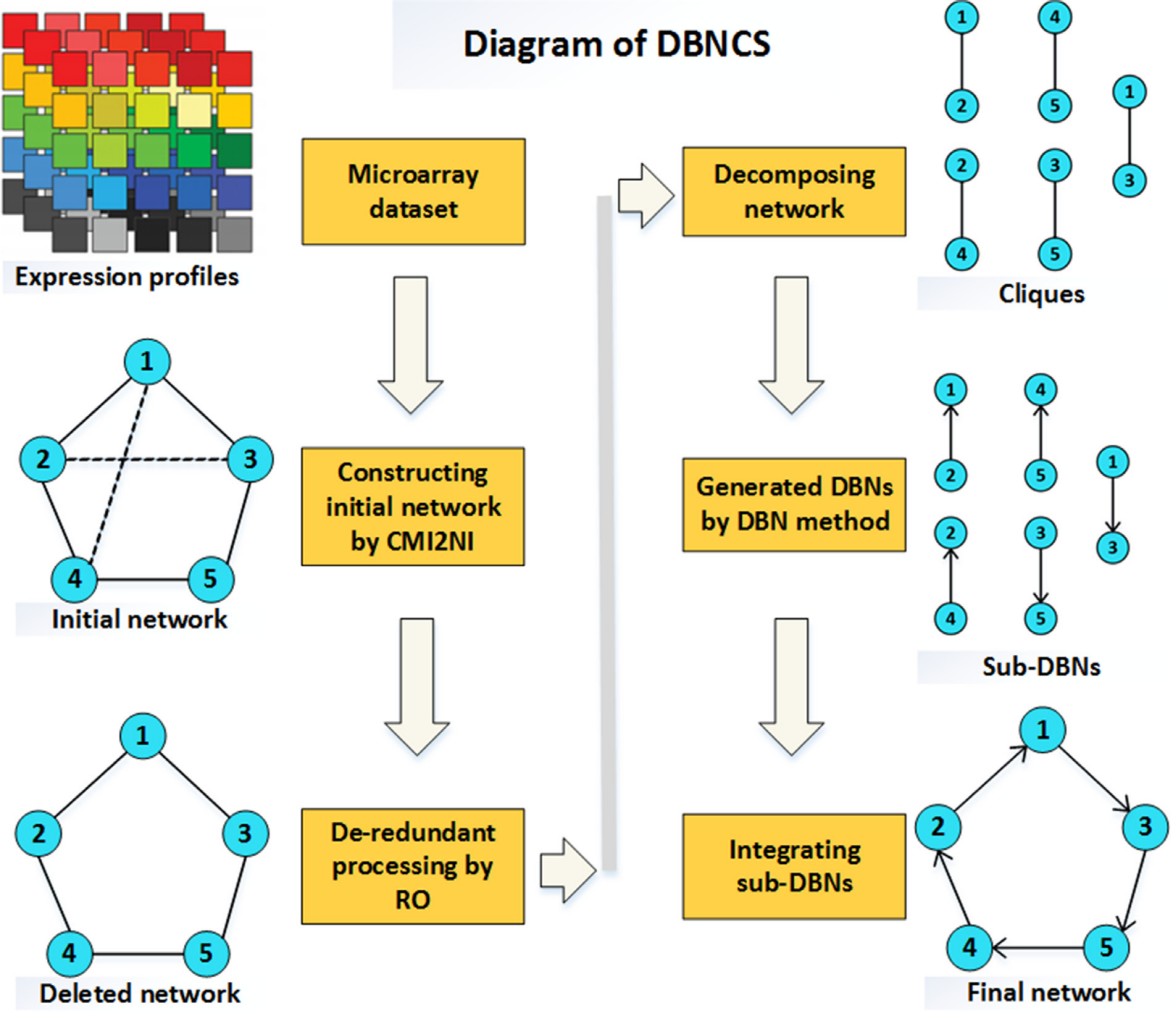
The diagram of DBNCS method (1) Using the CMI2NI algorithm, and through the gene microarray data to construct from the five genes and the interaction between the composition of the network structure profiles. (2) Redundant regulations are removed by RO algorithm: 2–3, 1–4. (3) The network structure profiles after the redundancy are decomposed into a series of cliques consisting of two co-expressing genes without loss: 2–1, 5–4, 1–3, 4–2, 3–5, the number of cliques is the same as the regulation relationship. (4) For each clique structure, the regulatory gene, target gene and the transcriptional delay are determined by the DBN based on the CS model to generate a series of dynamic sub-networks: 2→1,5→4,1→3,4→2,3→5. (5) Integration of a series of sub-networks, and get the final multiple time-delayed GRNs. The solid lines in Figure represent the true regulation between genes, and the dotted lines represent the redundancy correlation between the two genes.

In this paper, a hybrid learning method based on DBN to construct the multiple time-delayed GRNs is proposed, combining the comprehensive score (CS) with the DBN model. For convenience, this algorithm is called DBNCS. The DBNCS algorithm uses the CMI2NI algorithm to construct the network structure profiles, namely the search space, and uses the DBN model to identify the direction of gene regulation and search for the optimal network structure. Experimental environment: Intel (R) Xeon (TM) CPU E5-2650 @ 2.30GHz 2.30GHz with 32.0GB of RAM, MATLAB 2014b programming implementation. Figure 8 is the schematic diagram of our DBNCS method, which is described in detail as follows.

DBNCS algorithm to reconstruct multiple time-delayed GRNs specific implementation steps are as follows:

### Step 1 Construct the network structure profiles (CMI2NI) through CMI2

In general, a group of genes that have high CMI2 values are co-expressed genes, one of which is the target gene and the other is a regulatory gene. For gene expression datasets with *n* genes, a complete graph with *n* nodes is constructed first and set a decision independence threshold *θ*. Let *L* = −1, *L* is the maximum number of variable *Z* in Equation (8). Let *L* = *L* + 1, for each non-zero edge, G(i,j)≠0, and select genes from the network that are simultaneously linked to the genes *i* and *j*, and calculate the number of genes (not including genes *i* and *j*), recording as *T*. If *T* < *L*, termination algorithm, that is, the final network structure profiles is *G*. If *T* ≥ *L*, in order to reduce the computational complexity and ensure the accuracy of network inference, *L* genes are selected from *T*genes as conditional genes, and let them be $K=[k_{1},k_{2},\cdots ,k_{L}]$. *K* has $C_{T}^{L}$ kinds of selections, for each gene *L*, $C_{T}^{L}$ CMI2(i,j|K) are calculated by equation (8), select their maximum, recorded as $\mathrm{CMI}2_{\max}(i,j|K)$. If $\mathrm{CMI}2_{\max}(i,j|K)<\theta$, then let *G*(*i, j*) = 0. This process is repeated until no edge can be removed, so that the network structure profiles can be obtained, that is, building DBN search space to determine the independence of information between genes.

### Step 2 Redundant regulations are removed by the RO algorithm

In this step, only genes with coexpression relationships in the network structure profiles G can be removed redundant regulations by the RO optimization model. Taking the formula (9) as the objective function, we can calculate the regulation intensity vector *β^RO^*. Set the threshold *θ*_0_, the regulation relationship is less than *θ*_0_, the regulation relationship is determined as the low regulation intensity relationship, and the regulation intensity of the gene pairs are set to zero, and the nonzero variables in the model are reestimated in the next step. In this re-optimization solution, we can eliminate the effect of noise and redundant regulation to a certain extent, and reduce the false positive rate. In order to achieve the highest accuracy, the above process will be repeated until there is no non-zero variables needed to be reestimated so far, and finally get removal of redundant network structure profiles *G*′. A target gene is usually regulated by multiple transcription factors, RO optimization model can control the sparseness of the network by adjusting the coefficients.

### Step 3 The network structure profiles are decomposed into a series of cliques without loss

For a large GRN, searching for the optimal network structure among all possible structures using the DBN model is a NP-hard problem [87]. In order to avoid the above problems, DBNCS method uses CMI2NI algorithm to construct search space, and the lossless search space is decomposed into a series of two-phase regulated genes composed of a clique structue. As the clique contains only a few nodes, it can realize the greedy search of DBN model for network structure, while ensuring the accuracy of network construction accuracy and significantly reducing the computational complexity. Assuming that each gene is a potential target gene, the gene co-expressed in the network structure profile serves as a regulatory gene. Based on this assumption, the network structure profiles can be decomposed into *D* cliques, where *D* is the number of regulatory relations existing in the network structure profiles. Each clique consists of gene *G*_i_ and one co-expressed gene.

### Step 4 Determine the optimal network in the clique structure by DBN model

In the clique structure obtained in step 3, the transcription delay of gene pairs are determined according to the MI at different time delay, and a transcription time delay matrix *N*×*N* is generated, *N* represents the number of genes. Data reorganization is followed. The traditional BN model is characterized by its single feature, insufficient information mining, poor stability, and high computational cost to construct large-scale regulatory network. DBNCS algorithm applies the comprehensive score model to the DBN, and uses the DBN hybrid learning method to construct the GRNs within the cliques, thereby identifying the direction of gene regulation and significantly reducing the cost of computing. The comprehensive score model can extract the linear correlation, nonlinear correlation and dynamic characteristics among the gene expression data, fully excavate the regulatory information, realize the accurate measurement of the causal intensity among the genes, and reduce the false positives of the network construction. In each clique, each possible network structure is scored by the formula (13), that is, the DBN with the comprehensive score model is used for the greedy search, and the highest scoring network structure is selected as the optimal subnetworks, which identified the gene regulation direction, determined regulatory gene and target gene in the co-expression gene, and realized the transformation of the clique structure to the dynamic subnetworks.

### Step 5 Integrate dynamic subnetworks to get the final multiple time-delayed GRNs

Based on the stationarity assumption and the Markov assumption, all dynamic subnetworks are integrated into a directed network as the final multiple time-delayed GRNs, from which the regulation relationship and the transcriptional time delay can be understood. In the process of constructing subnetworks, DBNCS not only determined the regulatory genes and target genes between co-expression genes, but also determined the transcriptional delay of gene regulation. Among them, the prior network of multiple time-delayed GRNs is the network structure which is determined the regulatory direction by the comprehensive score function in the network structure profiles, and the transfer network is the corresponding transcription delay matrix of the network structure.

## CONCLUSIONS

As an important research field in system biology, GRNs explains complex life phenomena from the perspective of gene interactions, and attempts to understand the temporal and spatial mechanisms of GRNs by establishing regulatory model. In order to overcome the shortcomings of the information-based approach and the traditional Bayesian network construction method, this paper applies the comprehensive score model to the dynamic Bayesian network and we proposed a novel hybrid learning algorithm (DBNCS) based on DBN to construct the multiple time-delayed GRNs. The algorithm uses CMI2NI algorithm to study the network structure profiles, and uses the recursive optimization algorithm to remove the redundant regulations, thereby reduce the false positive rate of network construction. The CMI2 algorithm is used to calculate the optimal transcription time-delayed between pairs of genes in the search space. After the network structure profiles are decomposed into multiple cliques without loss, the DBN model is used to identify the direction of gene regulation within cliques, and the optimal network structure search is performed, which significantly reduces the computational complexity. Among them, the comprehensive score model not only uses the TRS algorithm to mine the multi-delay dynamic information in the gene expression data, but also through the recursive optimization algorithm mines linear correlation information, through CMI2 mining non-linear correlation information, and comprehensively considers the three aspects, which accurately calculate the causal intensity between genes, and effectively avoid the structural loss caused by the single model features. But in the absence of a priori information, such as the unknown real network, how to accurately determine the threshold of judgment independence is still an unresolved problem [88–90]. On the benchmark GRNs from DREAM3 challenge simulation data and a real data of SOS DNA repair network in E. coli, the simulation results confirmed the effectiveness of our method (DBNCS), which is superior to other state-of-the-art methods. Combining the Context Likelihood of Relatedness (CLR) algorithm [52] may get better results when performing threshold judgments, which will be our next research work. The source code for implementing in this study is available from the author upon request.

## References

[1] Davidson EH, Erwin DH (2006). Gene regulatory networks and the evolution of animal body plans. Science.

[2] Guo WB, Calixto CPG, Tzioutziou N, Lin P, Waugh R, Brown JWS, Zhang RX (2017). Evaluation and improvement of the regulatory inference for large co-expression networks with limited sample size. BMC Syst Biol.

[3] Teichmann SA, Babu MM (2004). Gene regulatory network growth by duplication. Nat Genet.

[4] Buckingham M, Rigby PW (2014). Gene Regulatory networks and transcriptional mechanisms that control myogenesis. Dev Cell.

[5] Kitano H (2002). Systems Biology: A brief overview. Science.

[6] Noble D (2002). Modeling the heart-from genes to cells to the whole organ. Science.

[7] Hiroaki K (2002). Computational systems biology. Nature.

[8] Duren Z, Chen X, Jiang R, Wang Y, Wong WH (2017). Modeling gene regulation from paired expression and chromatin accessibility data. Proc Nati Acad Sci USA.

[9] Wang Y, Joshi T, Zhang XS, Xu D, Chen LN (2006). Inferring gene regulatory networks from multiple microarray datasets. Bioinformatics.

[10] Wu J, Zhao XD, Lin ZL, Shao ZF (2016). Large scale gene regulatory network inference with a multi-level strategy. Mol BioSyst.

[11] Verduijn M, Peek N, Rosseel PMJ, Jonge E, Mol BAJM (2007). Prognostic Bayesian networks: I: rationale, learning procedure and clinical use. J Biomed Inform.

[12] Isci S, Dogan H, Ozrurk C, Otu HH (2014). Bayesian network prior: network analysis of biological data using external knowledge. Bioinformatics.

[13] Qiu P, Gentles AJ, Plevritis SK (2009). Fast calculation of pairwise mutual information for gene regulatory network reconstruction. Comput Methods Programs Biomed.

[14] Yamazaki K (2015). Accuracy of Bayesian latent variable estimation with redundant dimension. Statistics.

[15] Yamazaki K (2015). Asymptotic accuracy of Bayesian estimation for a single latent variable. Neural Networks.

[16] Maetschke SR, Madhamshettiwar PB, Davis MJ, Ragan MA (2014). Supervised, semi-supervised and unsupervised inference of gene regulatory networks. Briefings in Bioinformatics.

[17] Pe’Er D, Regev A, Elidan G, Friedman N (2001). Inferring subnetworks from perturbed expression profiles. Bioinformatics.

[18] Shmulevich I, Dougherty ER, Kim S, Zhang W (2002). Probabilistic Boolean networks: a rule-based uncertainty model for gene regulatory networks. Bioinformatics.

[19] Lähdesmäki H, Shmulevich I, Yli-Harja O (2003). On learning gene regulatory networks under the Boolean network model. Mach Learn.

[20] Liang J, Han J (2012). Stochastic Boolean networks: an efficient approach to modeling gene regulatory networks. BMC Syst Biol.

[21] Li P, Zhang CY, Perkins EJ, Gong P, Deng YP (2007). Comparison of probabilistic Boolean network and dynamic Bayesian network approaches for inferring gene regulatory networks. BMC Bioinformatics.

[22] Li Z, Li P, Krishnan A, Liu JD (2011). Large-scale dynamic gene regulatory network inference combining differential equation models with local dynamic Bayesian network analysis. Bioinformatics.

[23] Cao JG, Qi X, Zhao HY (2012). Modeling gene regulation networks using ordinary differential equations. Methods Mol Biol.

[24] Chen KC, Wang TY, Tseng HH, Huang CYF, Kao CY (2005). A stochastic differential equation model for quantifying transcriptional regulatory network in saccharomyces cerevisiae. Bioinformatics.

[25] Singh M, Valtorta M (1995). Construction of Bayesian network structures from data: a brief survey and an efficient algorithm. Internat J Approximate Reasoning.

[26] Cooper GF, Herskovits E (1992). A Bayesian method for the induction of probabilistic networks from data. Mach Learn.

[27] Chai LE, Mohamad MS, Deris S, Chong CK, Choon YW, Omatu S (2014). Current development and review of dynamic Bayesian network-based methods for inferring gene regulatory networks from gene expression data. Curr Bioinformatics.

[28] Liu ZP (2015). Reverse engineering of genome-wide gene regulatory networks from gene expression data. Curr Genomics.

[29] Tsamardinos I, Brown LE, Aliferis CF (2006). The max-min hill-climbing Bayesian network structure learning algorithm. Mach Learn.

[30] Friedman N, Koller D (2003). Being Bayesian about network structure. a Bayesian approach to structure discovery in Bayesian networks. Mach Learn.

[31] Yu J, Smith VA, Wang PP, Hartemink AJ, Jarvis ED (2004). Advances to Bayesian network inference for generating causal networks from observational biological data. Bioinformatics.

[32] Beal MJ, Falciani F, Ghahramani Z, Rangel C, Wild DL (2005). A Bayesian approach to reconstructing genetic regulatory networks with hidden factors. Bioinformatics.

[33] Cerulo L, Elkan C, Ceccarelli M (2010). Learning gene regulatory networks from only positive and unlabeled data. BMC Bioinformatics.

[34] De SR, Marchal K (2010). Advantages and limitations of current network inference methods. Nat Rev Microbiol.

[35] Heckerman D, Geiger D, Chickering DM (1995). Learning Bayesian networks: the combination of knowledge and statistical data. Mach Learn.

[36] Friedman N, Murphy K, Russell S (1998). Learning the structure of dynamic probabilistic networks. Computer Science.

[37] Sales G, Romualdi C (2011). Parmigene-a parallel R package for mutual information estimation and gene network reconstruction. Bioinformatics.

[38] Zhao J, Zhou YW, Zhang XJ, Chen LN (2016). Part mutual information for quantifying direct associations in networks. Proc. Nati. Acad. Sci. USA.

[39] Chen XW, Anantha G, Lin XT (2008). Improving Bayesian network structure learning with mutual information-based node ordering in the K2 algorithm. IEEE Trans Knowl Data Eng.

[40] Xiao F, Gao L, Ye YS, Hu YX, He RJ (2016). Inferring gene regulatory networks using conditional regulation pattern to guide candidate genes. Plos One.

[41] Chaitankar V, Ghosh P, Perkins EJ, Gong P, Zhang CY (2010). Time lagged information theoretic approaches to the reverse engineering of gene regulatory networks. BMC Bioinformatics.

[42] Wang K, Saito M, Bisikirska BC, Alvarez MJ, Lim WK, Rajbhandari P, Shen Q, Nemenman I, Basso K, Margolin AA, Klein U, Dalla-Favera R, Califano A (2009). Genome-wide identification of post-translational modulators of transcription factor activity in human B cells. Nat Biotechnol.

[43] Sumazin P, Yang X, Chiu HS, Chung WJ, Iyer A, Llobet-Navas D, Rajbhandari P, Bansal M, Guarnieri P, Silva J, Califano A (2010). An extensive microRNA-mediated network of RNA-RNA interactions regulates established oncogenic pathways in glioblastoma. Cancer Res.

[44] Zhang X, Zhao XM, He K, Lu L, Cao YW, Liu JD, Hao JK, Liu ZP, Chen LN (2012). Inferring gene regulatory networks from gene expression data by path consistency algorithm based on conditional mutual Information. Bioinformatics.

[45] Shannon P, Markiel A, Ozier O, Baliga NS, Wang JT, Ramage D, Amin N, Schwikowski B, Ideker T (2003). Cytoscape: A software environment for integrated models of biomolecular interaction networks. Genome Res.

[46] Adabor ES, Acquaah-Mensah GK, Oduro FT (2014). SAGA: a hybrid search algorithm for Bayesian network structure learning of transcriptional regulatory networks. J Biomed Informatics.

[47] Butte AJ, Kohane IS (2000). Mutual information relevance networks: functional genomic clustering using pairwise entropy measurements. Pac Symp Biocomput.

[48] Altay G, Emmert-Streib F (2011). Structural influence of gene networks on their inference: analysis of C3NET. Biolo Direct.

[49] Liu F, Zhang SW, Guo WF, Wei ZG, Chen L (2016). Inference of gene regulatory network based on local Bayesian networks. Plos Comput Biol.

[50] Frank ES, Glazko GV, Gökmen A, Ricardo DMS (2012). Statistical inference and reverse engineering of gene regulatory networks from observational expression data. Front Genet.

[51] Schaffter T, Marbach D, Floreano D (2010). GeneNetWeaver: in silico benchmark generation and performance profiling of network inference methods. Bioinformatics.

[52] Faith JJ, Hayete B, Thaden JT, Mogno I, Wierzbowski J, Cottarel G, Kasif S, Collins JJ, Gardner TS (2007). Large-Scale mapping and validation of Escherichia coli transcriptional regulation from a compendium of expression profiles. Plos Biology.

[53] Marbach D, Prillc RJ, Schafftera T, Mattiussia C, Floreanoa D, Stolovitzkyc G (2010). Revealing strengths and weaknesses of methods for gene network inference. Proc Nati Acad Sci USA.

[54] Marbach D, Costello JC, Küffner R, Vega NM, Prill RJ, Camacho DM, Allison KR, Kellis M, Collins JJ, Stolovitzky G, The DREAM5 Consortium (2012). Wisdom of crowds for robust gene network inference. Nature Methods.

[55] Marbach D, Schaffter T, Mattiussi C, Floreano D (2009). Generating realistic in silico gene networks for performance assessment of reverse engineering methods. J Comput Biol.

[56] Li Z, Li P, Krishnan A, Liu JD (2011). Large-scale dynamic gene regulatory network inference combining differential equation models with local dynamic Bayesian network analysis. Bioinformatics.

[57] Liu J, Chi YX, Zhu C, Jin YC (2017). A time series driven decomposed evolutionary optimization approach for reconstructing large-scale gene regulatory networks based on fuzzy cognitive maps. BMC Bioinformatics.

[58] Liu W, Zhu W, Liao B, Chen XT (2016). Gene regulatory network inferences using a maximum-relevance and maximum-significance strategy. Plos One.

[59] Xuan NV, Chetty M, Coppel R, Wangikar PP (2012). Gene regulatory network modeling via global optimization of high-order dynamic Bayesian network. BMC Bioinformatics.

[60] Smoot ME, Ono K, Ruscheinski J, Wang PL, Ideker T (2011). Cytoscape 2.8: new features for data integration and network visualization. Bioinformatics.

[61] Xiong H, Choe Y (2008). Structural sysrems identification of genetic regulatory networks. Bioinformatics.

[62] Margolin AA, Nemenman I, Basso K, Wiggins C, Stolovitzky G, Favera RD, Califano A (2006). ARACNE: an algorithm for the reconstruction of gene regulatory networks in a mammalian cellular context. BMC Bioinformatics.

[63] Ronen M, Rosenberg R, Shraiman BI, Alon U (2002). Assigning numbers to the arrows: parameterizing a gene regulation network by using accurate expression kinetics. Proc Nati Acad Sci USA.

[64] Guo X, Zhang Y, Hu W, Tan H, Wang X (2014). Inferring nonlinear gene regulatory networks from gene expression data based on distance correlation. Plos One.

[65] Omranian N, Eloundou-Mbebi JMO, Mueller-Roeber B, Nikoloski Z (2016). Gene regulatory network inference using fused LASSO on multiple data sets. Scientific Reports.

[66] Banf M, Rhee SY (2017). Computational inference of gene regulatory networks: approaches, limitations and opportunities. Biochim Biophys Acta-Gene Regul Mech.

[67] VânAnh HT, Alexandre I, Louis W, Pierre G (2010). Inferring regulatory networks from expression data using tree-based methods. Plos One.

[68] Zhang XJ, Liu KQ, Liu ZP, Duval B, Richer JM, Zhao XM, Hao JK, Chen LN (2013). Narromi: a noise and redundancy reduction technique improves accuracy of gene regulatory network inference. Bioinformatics.

[69] Tsamardinos I, Aliferis CF, Statnikov AR Algorithms for large scale Markov blanket discovery.

[70] Margaritis D, Thrun S (1999). Bayesian network induction via local neighborhoods. Advances in Neural Information Processing Systems.

[71] Zhang XJ, Zhao J, Hao JK, Zhao XM, Chen LN (2014). Conditional mutual inclusive information enables accurate quantification of associations in gene regulatory networks. Nucleic Acids Res.

[72] Jensen FV, Nielsen TD (2008). Bayesian networks and decision graphs. Statistics for Engineering and Information Science.

[73] Stolovitzky G, Monroe D, Califano A (2007). Dialogue on reverse-engineering assessment and methods. Ann N Y Acad Sci.

[74] Shen-Orr SS, Milo R, Mangan S, Alon U (2002). Network motifis in the transcriptional regulation network of escherichia coli. Nat Genet.

[75] Mengshoel OJ (2010). Understanding the scalability of Bayesian network inference using clique tree growth curves. Artificial Intelligence.

[76] Mengshoel OJ, Wilkins DC, Roth D (2006). Controlled generation of hard and easy Bayesian networks: impact on maximal clique size in tree clustering. Artificial Intelligence.

[77] Wang RS, Jin GX, Zhang XS, Chen LN (2009). Modeling post-transcriptional regulation activity of small non-coding RNAs in Escherichia coli. Bioinformatics.

[78] Liu W, Zhu W, Liao B, Chen HW, Ren SQ, Cai LJ (2017). Improving gene regulatory network structure using redundancy reduction in the MRNET algorithm. Rsc Advances.

[79] Yalamanchili HK, Yan B, Li MJ, Qin J, Zhao ZY, Chin FY, Wang JW (2014). DDGni: dynamic delay gene-network inference from high-temporal data using gapped local slignment. Bioinformatics.

[80] Wang YXR, Huang HY (2014). Review on statistical methods for gene network reconstruction using expression data. J Theor Biol.

[81] Zou M, Conzen SD (2005). A new dynamic Bayesian network (DBN) approach for identifying gene regulatory networks from time course microarray data. Bioinformatics.

[82] Li YF, Chen HF, Zheng J, Ngom A (2016). The max-min high-order dynamic Bayesian network for learning gene regulatory networks with time-delayed regulations. IEEE/ACM Transactions on Computational Biology and Bioinformatics.

[83] Dondelinger F, Lèbre S, Husmeier D (2013). Non-homogeneous dynamic Bayesian networks with Bayesian networks with Bayesian regularization for inferring gene regulatory networks with gradually time-varying structure. Mach Learn.

[84] Penfold CA, Buchananwollaston V, Denby KJ, Wild DL (2012). Nonparametric Bayesian inference for perturbed and orthologous gene regulatory networks. Bioinformatics.

[85] Chai LE, Loh SK, Low ST, Mohamad MS, Deris S, Zakaria Z (2014). A review on the computational approaches for gene regulatory network construction. Comput Biol Med.

[86] Liu ZP (2015). Reverse Engineering of genome-wide gene regulatory networks from gene expression data. Curr Genomics.

[87] Karpas E, Shimony SE, Beimel A (2009). Approximate belief updating in max-2-connected Bayes networks is NP-hard. Artificial Intelligence.

[88] Gowda T, Vrudhula S, Kim S (2009). Modeling of gene regulatory network dynamics using threshold logic. Ann N Y Acad Sci.

[89] Chen YH, Yang CD, Tseng CP, Huang HD, Ho SY (2015). GeNOSA: Inferring and experimentally supporting quantitative gene regulatory networks in prokaryotes. Bioinformatics.

[90] Stewart AJ, Seymour RM, Pomiankowski A, Plotkin JB (2012). The population genetics of cooperative gene regulation. BMC Evol Biol.

